# Vulnerability as determinant of suicide among older people in Northern Indian states

**DOI:** 10.3389/fpsyg.2022.971080

**Published:** 2022-09-15

**Authors:** Avanish Bhai Patel

**Affiliations:** Alliance School of Liberal Arts, Alliance University, Bengaluru, India

**Keywords:** document analysis, older people, suicide, vulnerability, India

## Abstract

Older people are confronted with a myriad of challenges throughout the course of their lives in the present society. One of these is the issue of suicidal behaviour among people of older age. This article understands the nature and examines the cause of mortality due to suicide among older people in later life. The author has applied the document analysis method. The information for the current research has been collected using the news content of various Indian newspapers, magazines, and news portals. The researcher was collated 60 occurrences of old-age suicide from the newspapers of Northern Indian states from March 2022 to June 2022. The study has indicated that there is a substantial rise in old-age suicide due to the vulnerable condition of older people in late life. Moreover, the vulnerable condition of older people has led to various factors instrumental in old-age suicide, such as abuse in the family, chronic diseases, depression, poverty, and social rejection that give rise to feelings of committing suicide among the older people.

## Introduction

The population of older people in the world has grown steadily during the last years of the last century. The number of people aged 60 years and over in the world was 673 million in the early years of the 21st century and is expected to increase to 2 billion by 2050 (Shettar, 2013; Kumar and Rathour, 2019). Nearly, one-fifth of the population in more developed countries is over 60, compared to 8% in developing regions (Shettar, 2013; Kumar and Rathour, 2019). Similar to many other countries, India is now observing an increase in the proportion of its aged population (Rajan et al., 2003; Kalavar et al., 2013; Shettar, 2013). In the last 50 years, the Indian population has almost tripled, while the elderly population has expanded by more than fourfold. The number of people aged 60 years and older in India is projected to climb from 100 million in 2011 to 179 million in 2031 and then to 315 million in 2050, which represents a significant increase from the 2011 figure of 100 million (Rajan et al., 2003; Kalavar et al., 2013). The socio-economic and demographic situation of the country is constantly shifting, and as a result, significant changes are being observed in the living conditions of older people throughout the country (Raju, 2011). Not only the number of older people in the country is increasing but also their life expectancy is reaching new heights on an annual basis because of developments in medical technology, improvements in the quality of life, and general progress made in the country as a whole (Rajan et al., 2003; Raju, 2011). As the population of older people keeps increasing, the number of problems they have is also increasing quickly. The problem of suicide is one of them among older people.

The death of older people caused by suicide is a matter of grave concern across all Indian states in contemporary times. At present, a number of older people in Indian society are taking their own lives because of their vulnerable conditions in later life. Suicide among older people has not been a problem in India because of its congenial social environment and value-based family structure. However, it has been pointed out that the roots of a congenial social environment and value-based family structure are eroding very fast due to changing socio-economic scenarios, such as industrialisation, urbanisation, and globalisation (Jamuna, 2000; Khan, 2004; Raju, 2011). The rapidly increasing number of aged people is compounded by the disintegration of value-based familial structure and the ever-increasing influence of socio-economic changes and new lifestyles. The care for older people has emerged as an important issue in India (Jain, 2008; Shettar, 2013). In such changing situations, the majority of the elderly, who have passed most of their life with their joint families, are on the verge of isolation, alienation, hopelessness, and depression in old age (Khan, 2004). When they need maximum family and social support, they live alone and feel neglected. Sometimes, older people are abused in the family and society. The coping capacities (interpersonal relationships) of older people are now being challenged under various circumstances, causing vulnerable conditions for them in many ways, such as low belongingness and perceived burdensomeness. Consequently, suicide among older people in Indian society is emerging as a social problem.

**FIGURE 1:**
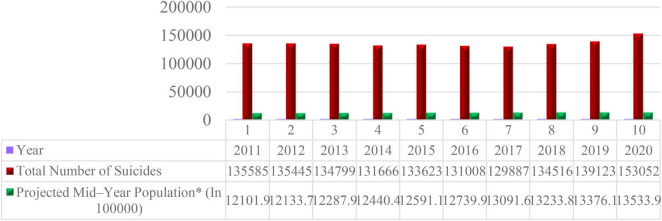
Trend of overall suicide in India from 2010 to 2020. Source: National Crime Record Bureau [NCRB] (2020).

As the proportion of older people in the population will increase all over the world in the coming decades, the cases of suicide among older people are also expected to be on the rise consequently (Bharati, 2021). World Health Organization [WHO] (1992) pointed out that their vulnerable condition due to low belongingness and perceived burdensomeness in late life implies a significant suicide risk as compared to any other age groups. Suicide ranks in the top 10 causes of death among older people (Blazer et al., 1986; Bharati, 2021). The reason behind more suicides among older people is found that older people face many problems, such as physical, psychological, social, and economic problems in later life, which contribute to increased vulnerability among older people (Behera et al., 2007; Bharati, 2021). In 2019, it was reported globally that 14.25% of people per 100,000 people aged 50–69 years committed suicide, and 24.53% of people per 100,000 people aged 70 years or older did the same (Ritchie et al., 2019). It is reported by the National Crime Record Bureau [NCRB] (2020) in its yearly nationwide survey on suicide that around 1,358,704 people committed suicide from 2011 to 2020 in India (Figure 1). National Crime Record Bureau [NCRB] (2020) reported in its annual report on suicide in the country that India has recorded 153,052 cases of suicide, which reports around 418 cases of suicide daily in India. National Crime Record Bureau [NCRB] (2020) indicated that the suicide rate per 100,000 population also increased from 10.4% in 2019 to 11.3% in 2020. National Crime Record Bureau [NCRB] (2020) reported in its yearly report that around 13,126 older people per 100,000 population committed suicide in India. According to National Crime Record Bureau [NCRB] (2020), abuse, alcohol addiction, chronic diseases, family issues, feelings of loneliness, financial loss, mental illnesses, and other factors contribute to suicide among older people in India.

In the last few years, the problem of suicide among older people has attracted social scientists from different regions of the country. Many studies (Tanuj et al., 2009; Shah and Escudero, 2017; Gover et al., 2020; Padmaja and Bharadwaj, 2020; Rana, 2020; Bharati, 2021; De Sousa, 2021; Sourabh et al., 2021) related to suicide among older people have been done and are being done in India. Through these studies, the nature, causes, and effects of suicide have been studied scientifically and are seen as a serious problem among older people in India. It is apparent from these studies that suicide among older people is a matter of grave concern in contemporary Indian society because it is the worst form of death. Many studies on suicide among older people have been conducted from different perspectives to understand the causes and consequences of the suicidal tendency among older people in India. However, these studies have not looked at the causes of suicidal tendencies among older people from a vulnerability approach. In this backdrop, this article attempts to analyse the determinants of suicide among older people through a vulnerability approach. It accesses the nature and magnitude of suicide among older people in India and aims to understand the subjective meaning behind the social fact of suicide among older people.

## Vulnerability approach to suicide among older people

**Diagram 1:**
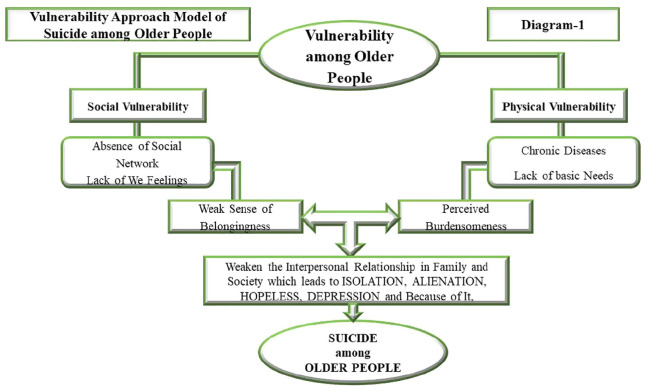


Vulnerability states the diminished capacity of an individual who feels that he does not have a strong social and physical ability to protect himself from any problem (personal, family, and social problems) (Hale, 1996; Vandeviver, 2011). There are four groups, namely, older people, women, poor, and ethnic minorities, who feel vulnerable themselves and also confront more problems (personal, family, and social problems) due to their vulnerability (Powell and Wahidin, 2007; On Fung et al., 2009). The vulnerability approach explains the vulnerable conditions of older people due to frailty or weakness, which lead to various problems among them, in which suicide is one of them (Sarvimaki and Hult, 2014). Being vulnerable due to frailty or weakness refers to the kind of vulnerability among older people that is usually associated with social vulnerability and physical vulnerability (Sarvimaki and Hult, 2014). The vulnerability approach of older people tries to point out negative attitudes due to many factors, such as social and physical factors in later life that lead to suicidal tendencies (Bharati, 2021). On the one hand, the social vulnerability of older people talks about the absence of social networks (attachment, involvement, commitment, and belief) that weaken the sense of belongingness between the older people and family members. On the other hand, the physical vulnerability of older people talks about the poor conditions (illness, lack of care, and basic needs) among them that are perceived as burdensomeness by their family members. Both a weak sense of belongingness and perceived burdensomeness are closely related to the interpersonal relationships of a person and their contribution to suicidal tendency (Joiner, 2005). The nature and problem of suicide among older people in India can be explained and examined by using the vulnerability approach that has been presented in Diagram 1.

Patel and Mishra (2018) pointed out that older people believe they are socially vulnerable due to the fact that as they age, they become less involved in social activities and complain that people do not have time for older people and that they do not have a “we feeling” like people did in the past. All these feelings produce isolation and alienation among older people. The materialistic way of life has also had a significant impact on social structure, and it has done its part to contribute to the worsening of the psychological and social issues that affect older people (Patel and Mishra, 2018). Older people have been left abandoned, uncared for, and lonely as a result of the rise of nuclear families, working couples, and changes in the behaviour of residents in the neighbourhood. The social support of older people has weakened. As a consequence of this, a significant number of elderly individuals are feeble, vulnerable, and unable to help themselves. Due to these factors, elderly adults are more likely to experience feelings of loneliness and isolation, and their sense of connection to their families is diminished. Consequently, older people commit suicide.

Patel and Mishra (2015) found that older people suffer from many physical problems in later life, such as chronic diseases and basic needs. Older people are unable to participate in physically active pursuits because of physical problems, and they must rely on the assistance of other members of their families. As a result of their physical ailments and basic requirements, older people often realise that they are a burden to their family members. Older people, upon coming to terms with the burden of their responsibilities, conclude that their passing will bring their families greater happiness than their continued existence (Joiner, 2005). Consequently, more individuals commit suicide as they grow older.

## Document analysis method

**Diagram 2:**
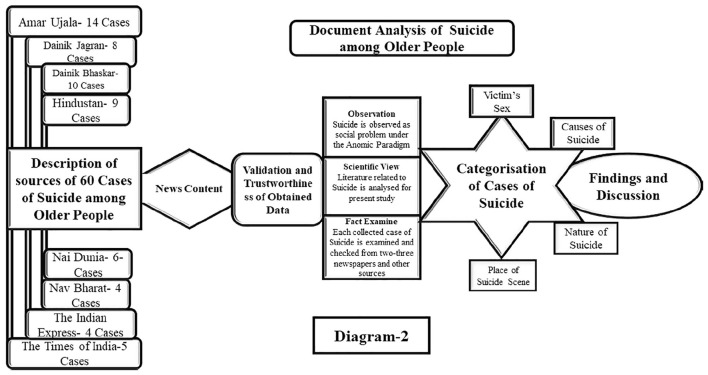


The researcher has used the document analysis method, which is basically a kind of content analysis, for the study of suicide among older people in India. The aim of the document analysis was to investigate and analyse any issue that is printed and electronic documents in the form of books, newspaper articles, and magazines (Bowen, 2009). “Document analysis necessitates the examination and interpretation of data to elicit meaning, gain comprehension, and develop scientific knowledge” (Corbin and Strauss, 2008; Bowen, 2009). Basically, in a document analysis study, a researcher utilises quantitative and qualitative analyses to identify the numerous words and ideas in a text, as well as their meaning and connections, and then draw conclusions about the text (Sarantakos, 1998; Sahoo and Patel, 2021). The author of this research mostly relied on secondary data that were gathered from various newspapers (both print and electronic) and magazines based in India. The old-age suicide data for the study have been collected from famous daily Hindi and English language newspapers, such as Amar Ujala, Dainik Bhaskar, Dainik Jagran, Hindustan, Nav Bharat, Naidunia, The Indian Express, and The Times of India. Moreover, the author has also collected old-age suicide cases from news portals, such as ETV Bharat, TV9, News 18, and NDTV. The data were collected from March 2022 to June 2022. The process of document analysis is illustrated in Diagram 2.

The data on old-age suicide have been obtained from Northern states of India, such as Bihar, Delhi, Haryana, Madhya Pradesh, Maharashtra, Rajasthan, Uttar Pradesh, and Uttarakhand. In view of the fact that instances of suicide committed by older people are often reported and highlighted by newspapers and other news portals in these states. This study’s sample cases were acquired manually by reading every suicide-related news article published in newspapers. The researcher discovered typical keywords used by Indian journalists while reporting on a certain topic in newspapers and utilised those keywords to search for information on Internet platforms. The terms such as suicide, suicidal propensity, self-harm, self-killing, chronic illnesses, depression, mental health, and frustration, among others are utilised while doing a web search for information about suicide in India.

Therefore, the researcher has decided to conduct a study on old-age suicide in these states. The author collected around 100 newspaper articles on old-age issues using document analysis. From these 100 news articles, the author chose 60 news articles related to old-age suicide. This study’s sample cases were acquired manually by reading every suicide-related news article published in newspapers. The majority of the 60 reported cases of old-age suicide were found in these regions from the age group of 60 years to above 80 years. Each of these incidents of elder suicide has been divided into several themes, such as gender (male and female), causes of suicide among older people (chronic disease, depression, abuse, poverty, and social rejection), and nature of suicide (personal problem-based suicide, family problem-based suicide, and social problem-based suicide). Thus, the researcher has gathered data on old-age suicide from both print and electronic media, and the author has used document analysis to describe the nature and causes of old-age suicide in India.

The problem of suicide is very sensitive in India and people associate suicide with stigma. For this reason, the family members of the affected person do not openly talk about the cause of committing suicide. Due to this, a researcher has to face many difficulties in conducting a primary survey on suicide. Apart from this, the data on suicide collected by the National Crime Record Bureau in India do not show rural–urban differences in suicide in its report. Moreover, N.C.R.B. does not separately publish the causes of old-age suicides in its suicide data, which does not make the understanding of the nature of suicide among older people. For this reason, when a researcher wants to study suicide focused on older people, he does not get the data related to rural–urban differences, causes, and nature of suicide among older people. However, when a researcher collects data on suicide through newspapers, he gets the data easily whatever he wants to examine. Consequently, this method of data collection is chosen as the most suitable for gathering data on old-age suicide within the stipulated time frame. The incidents of old-age suicide have been meticulously collated from news items published in different Indian newspapers and news portals. Using observation, scientific view, and fact examination, the researcher has analysed each instance from two or three newspapers and other sources (Sahoo and Patel, 2021). This has assisted the research in proving the validity of obtained data on old-age suicide and assuring the reliability of data collected.

## Findings

### Place of suicide scene

Table 1 reveals that a total of 37 (61.67%) cases of old-age suicide have been recorded in urban areas, compared to 23 (38.33%) cases of old-age suicide in rural areas. The suicide rate is often reported to be greater in urban areas due to a multitude of urban pressures, such as overcrowding and social isolation (Radhakrishnan and Andrade, 2012).

**TABLE 1:** Place of suicide scene.

Place of suicide scene	Frequency	Percentage (%)
Rural	23	38.33
Urban	37	61.67
Total	60	

### Victims’ sex

Table 2 shows that 34 (56.67%) old men and 26 (43.33%) old women have killed themselves, respectively. The data show a major gap in the number of suicide deaths between the genders. According to the data (National Crime Record Bureau [NCRB], 2020), in 2020, while 3,300 old women died by suicide, the figure for old men was 74.85%, with 9,826 deaths by suicide. A study reveals that men continue to suffer discreetly from mental illness more than women, concealing that social stigma plays a significant role in men committing suicide (Ghosh, 2021).

**TABLE 2:** Victim’s sex.

Victim’s sex	Frequency	Percentage (%)
Male	34	56.67
Female	26	43.33
Total	60	

### Causes of suicide

**FIGURE 2:**
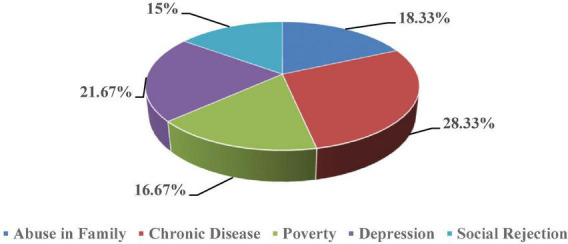
Causes of suicide.

Figure 2 demonstrates that most suicides (28.33%) have been caused by chronic disease, followed by several other causes of suicide, such as depression (21.67%), abuse in family (18.33%), poverty (16.67%), and social rejection (15.00%).

### Nature of suicide among older people

**FIGURE 3:**
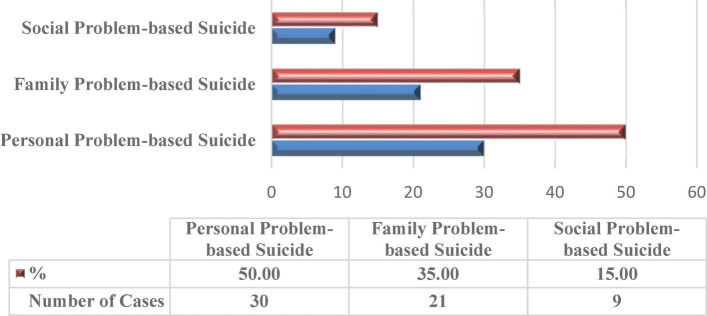
Nature of suicide among older people.

This study (Figure 3) has found that vulnerable conditions of older people have arisen in three natures of suicide, namely, personal problem-based suicide, familial problem-based suicide, and social problem-based suicide. The study has found that most cases of suicide have been committed due to personal problems (50.00%), followed by familial problems (35.00%) and social problems (15.00%).

### Personal problems-based suicide

Personal anomie refers to a condition in which personal problems arise before an individual in society due to the effect of rapid social change and unwanted phenomena. When the person is unable to overcome the problems arising due to these side effects, he is compelled to commit suicide. This study examines the nature of suicide among older people as motivated by personal causes. The researcher has pointed out in his study that personal problems were the underlying cause of suicide in 50.00% of cases. The researcher has found through analysis of news content that older people who had a personal nature of suicide were more likely to suffer from problems, such as chronic disease (28.33%) and depression (21.67%).

For example, based on news content, we may observe two significant suicide occurrences in which an old person took his or her own life due to chronic disease and depression.

### Familial problems-based suicide

Familial problems may be defined as any kind of dysfunction within a family due to rapid change and irrelevant incidents. In such a situation, a person does not live up to his or her family’s expectations, and his or her family situation becomes unstable. When the person does not see a way out of the family problems, he is forced to commit suicide. The researcher has found in his study that in approximately 35.00% of older people, the nature of suicide was due to familial problems. The study has found that older people who committed suicide were abused (18.33%) by family members and they lived in poverty (16.67%). For instance, based on news content, we may identify two prominent suicide occurrences in which an old person committed suicide as a result of abuse in family and poverty.

### Social problem-based suicide

Social problems are a condition of society in which due to rapid social change and irrelevant phenomena, social norms and values weaken and give rise to many problems. These problems promote a state of deviance affecting the whole society directly and indirectly. In such a situation, when the particular person who is suffering from it is unable to get out of these problems, he is forced to commit suicide. The researcher has found in the study that in the changing socio-economic scenario, approximately 15.00% of the total suicides of older people were committed due to social problems. When the researcher studied suicide among older people based on social problems, it was found that those who committed suicide were more likely to suffer from social rejection (15.00%). For example, we can discuss a case of suicide in which an old person committed suicide due to social rejection in the neighbourhood.

## Discussion

There is strong agreement that old age is a time of increasing vulnerability (Joseph and Cloutier-Fischer, 2005; Grundy, 2006; Schroder-Butterfill and Marianti, 2006; Crooks, 2009; Wiersma and Koster, 2013). It is the perception that old age connected with vulnerability is strongly linked to normative expectations of poor health and the increasing need for healthcare as people get older (Joseph and Cloutier-Fischer, 2005; Wiersma and Koster, 2013). Old age is also accompanied by a variety of social and economic changes, including the loss of employment, reduced income, and widowhood for many people (Arber et al., 2003; Crooks, 2009). The combination of these numerous age-related changes contributes to the perception of old age by many as a period of risk and uncertainty. However, older people differ greatly in their biological, physiological, psychological, and social circumstances (Crooks, 2009), laying the foundation for the study of differential vulnerability in old age. In the majority of vulnerability studies, the older people are consistently portrayed as high-risk populations. Vulnerability indicates the possibility of poor results because older people are unable to protect their wellbeing in later years (Crooks, 2009). Consequently, it is considered that older people as a vulnerable group face many social-psychological problems that lead to low belongingness and perceived burdensomeness constituting risk factors, such as suicide among older people.

Rapid socio-economic changes in the era of industrialisation, urbanisation, and globalisation have had positive and negative impacts on human beings and society. On the one hand, the positive impact has given a better life in society. On the other hand, the negative impact has weakened interpersonal relationships among family members. The weakened interpersonal relationships due to these socio-economic changes have made a vulnerable condition for older people in the family. The increasing incidents of suicide among older people are the result of this vulnerable condition. At present, all the suicides among older people happening in Indian society are directly or indirectly due to vulnerability in later life. This study has found that older people committed suicide due to social vulnerability and physical vulnerability. Moreover, during the COVID-19 pandemic, people avoided older people in the family and society because they were afraid of fear of contracting the coronavirus (Surjit, 2022). This made the lives of older people worse. Several newspapers have reported that people living in the same house were shunned in the family and society because of the COVID-19 pandemic. It excluded emotionally older people from their families and society as a whole. Older people were abused emotionally and physically in their own homes by their own children during the pandemic (Helpage India, 2021). The researcher has pointed out through news content that older people who committed suicide were suffering from personal problems (chronic disease and depression), family problems (abuse and poverty), and social problems (social rejection). All these problems affected their social bonding, resulting in social vulnerability and physical vulnerability. This study also points out that the rapid changes have featured social integration and regulation, resulting in low belongingness and perceived burdensomeness in the family and society due to the vulnerability of old people. The effect of low belongingness and perceived burdensomeness contribute to isolation, loneliness, hopelessness, and depression which, in turn, leads to old-age suicide in three ways, namely, personal problem-based suicide, familial problem-based suicide, and social problem-based suicide.

## Conclusion

This study is based on a content study of a few Hindi and English language Indian newspapers. Only a few states of India are included in the study’s sample regions, such as Bihar, Delhi, Haryana, Madhya Pradesh, Maharashtra, Rajasthan, Uttar Pradesh, and Uttarakhand. The chosen sample region does not provide a full picture of suicide among older people in India. The study suggests that suicide among older people is an alarming societal problem in contemporary times that may have been caused by a number of factors, such as social vulnerability (abuse in family, poverty, and social rejection) and physical vulnerability (chronic disease and depression) of older people in later life, which led to low belongingness and perceived burdensomeness. According to the data collected from March 2022 to June 2022, older men (56.67%) committed the majority of suicides. In addition, the study indicates that respect, honour, prestige, and authority held by older people in traditional Indian society have begun to decline progressively. In a society undergoing such rapid changes, older people are subject to social-psychological issues, such as loneliness, alienation, helplessness, and despair. All these social-psychological problems have a negative impact on the wellbeing of older people that contributes to the rising suicidal tendency among older people.

Finally, it is of the utmost significance to comprehend that our parents and other senior people are analogous to trees; they play a role in our courtyard, similar to that of providing shade and protection. They also provide us with the benefit of their knowledge and blessings due to that we go ahead smoothly in the journey of life. We owe a debt of gratitude to our elders who have shaped our personality, architected our vision of life, and taught us the ability to keep working after they have passed away. It should be not only our emotional responsibility but also our moral responsibility to provide a friendly environment for our parents and other older people at the end of their lives so that they can fully enjoy their lives without any problem.

## Data availability statement

The original contributions presented in this study are included in the article/supplementary material, further inquiries can be directed to the corresponding author.

## Author contributions

The author confirms being the sole contributor of this work and has approved it for publication.
